# Psychosocial Problems among School Going Adolescents in Nepal

**DOI:** 10.1155/2018/4675096

**Published:** 2018-07-02

**Authors:** Mina Timalsina, Mana Kafle, Rekha Timalsina

**Affiliations:** ^1^Padma Kanya Multiple Campus, Bagbazar, Kathmandu, Nepal; ^2^Patan Academy of Health Sciences, School of Nursing and Midwifery, Lalitpur Nursing Campus, Sanepa, Lalitpur, Nepal

## Abstract

**Background:**

Psychosocial problems refer to the difficulties faced by adolescents in different areas of personal and social functioning. Adolescents are vulnerable to psychosocial problems because of physical and physiological changes that occur in their body during this developmental stage. Therefore, this study was conducted to identify psychosocial problems among school going adolescents in Nepal.

**Methods:**

A cross-sectional descriptive study was adopted. Nonprobability convenient sampling technique was used for selecting 287 adolescents. Ethical approval was taken from Nepal Health Research Council and self-administered structured questionnaire was used for data collection. Data collection was done in 2016. Descriptive statistics and chi-square test were used to analyze the data.

**Results:**

The findings of this study show that 12.9 percent of adolescents had psychosocial problems. While categorizing psychosocial problems, the adolescents had internalizing problems (44.6%), attention deficit hyperactive disorder (ADHD) (25.8%), and externalizing problems (4.2%). There is association of age group and parent's marital status with psychosocial problems.

**Conclusion:**

It is concluded that psychosocial problems (i.e., internalizing problems, ADHD, and externalizing problems) were prevalent among Nepalese school adolescents. Adolescents' age group and parent's marital status are associated with psychosocial problems. This study recommended that school authority, health professionals, and other professional related to child health and mental health should play an important role for the prevention and earlier recognition of and intervention for psychosocial problems.

## 1. Introduction

Adolescence is the period of age ranging from 10 to 19 years, is one of the critical transitions in the lifespan that occurs after childhood and before adulthood, and is characterized by a tremendous pace in growth and change that is second only to that of infancy [1]. According to census 2011, age group from 10 to 19 years constituted 24.19 percent of the total population where 3207821 (12.11%) were male and 3199583 (12.08%) were female [2]. Psychosocial problems, such as behavioural, emotional, and educational problems are highly prevalent among children and adolescents [3]. Adolescents are vulnerable to psychosocial dysfunction when they suffer from physical injuries, psychological trauma, or major changes in their environments especially in the absent of strong support system [4]. Adolescence period is critical times for developing good mental health [5, 6]. Mentally healthy adolescents enjoy a positive quality of life; are free of symptoms of psychopathology; and function well at home, in school, and in their communities [7].

Lifetime psychiatric disorders usually have their first onset at a young age: half of them by 14 years and three-quarters by 24 years [5, 6]. Psychosocial problems have emerged as a threat in their overall development of adolescents [8]. A study in Dehradun showed that the overall prevalence of psychosocial problems was 40.5% [9]. Similarly, another cross-sectional study in Dehradun revealed that the overall prevalence of psychosocial problems among the adolescents was found to be 31.2% [10]. Similarly, a study in Nepal also revealed that 17.03% of adolescents were suffering from psychosocial dysfunction [11]. Adolescents have very special and distinct needs, which can no longer be overlooked. It is essential to invest in adolescent, as they are the future of the country. Therefore, this study was initiated an attempt for identifying the psychosocial problems among school going adolescents in Nepal. The results of this study are expected to contribute to design preventive programs, primarily focusing on psychological intervention for improving mental health of the school adolescents.

## 2. Research Methodology

### 2.1. Study Design

In December 2016, cross-sectional descriptive study was carried out for identifying the psychosocial problems of school going adolescents in Nepal.

### 2.2. Study Setting and Population

Study settings were three schools, namely, Nabingram Secondary School, Chapabot Higher Secondary School, and Shree Kalika Saran Higher Secondary School. These schools are located at Jarsingh Pauwa, ward no. 5 of Shankarapur municipality, Kathmandu, Nepal. The study population was all students (i.e., 402) from class 8^th^, 9^th^, and 10^th^ including boys and girls of that selected schools.

### 2.3. Sample Size and Sampling

Based on the students' presence at the time of data collection, 292 students were approached. Excluding the questionnaire having missing data, a total of 287 responses, i.e., 71.3% of target population, were used for the final analysis. Convenient sampling technique was adopted for the selection of those samples.

### 2.4. Data Collection Tools

The instrument used in this study was composed of two parts. Part one was related to questionnaire regarding sociodemographic information. Name of school, age, grade, religion, ethnicity, types of family, parent's educational status, occupational status, and marital status were included in this part.

Part two was a tool of the youth report of Pediatric Symptom Checklist (Y-PSC) [12] that was used to screen the psychosocial problem among adolescents. It was completed by the adolescent aged 11 and up. It consisted of 35 items that are rated as “Never,” “Sometimes,” or “Often” present and scored 0, 1, and 2, respectively. The total scores were calculated by adding together the score for each of the 35 items. Items which were left blank were simply ignored (i.e., score equals 0). If four or more items left blank, the questionnaire was considered invalid. The cutoff score for the Y-PSC was 30 or higher. For adolescents of all ages, a score of 30 or higher indicated significant psychological impairment. A positive score on the Y-PSC suggested the need for further evaluation by a qualified health or mental health professional [13].

For ensuring content validity, these instruments were further evaluated by consulting subject expert, advisor, experienced professionals, academics in child health and development, and psychologist to determine whether the instrument reflects the known content area. Reverse translation of the instruments was done. Pretesting was done among 25 students of Sarada secondary school by using Nepalese version of the questionnaire. It was done in order to establish the conceptual/linguistic and functional equivalence before the administration of the instruments to the actual sample of the study. Those students were similar in characteristics with actual samples of this study. Reliability coefficient of the Y-PSC was 0.808.

### 2.5. Data Collection Procedure

The duration of data collection was 2 weeks. It started from 18^th^ December 2016 to 29^th^ December 2016. Firstly, appointment for data collection was taken from the principal and class teacher. By considering ethical procedure, data collection was done from students in their respective classroom. Before distributing the questionnaire, orientation about the tools was given to the students and they were informed about the importance of responding to each statement of the questionnaire very carefully. Then, structured self-administered questionnaires regarding sociodemographic information and Youth Pediatric Symptoms Checklist were distributed to each respondent separately. Respondents took 20 minutes time in average for the completion of the questionnaire. Field editing and central editing were done for identifying its completeness, correctness, and accuracy at that time.

### 2.6. Data Analysis Procedure

The obtained data were edited, classified, and the different variables of the questionnaire were coded and double checked. Double data entry as well as data cleaning was done using Epi Data software. Then, the data were transferred to statistical package for social sciences (SPSS) software version 16. Descriptive statistics was used to describe the sample characteristics. For analyzing the association between sociodemographic characteristics and psychosocial problems among adolescents, chi-square test was used. For this test, significance was considered at* p *≤ .05 for 95% confidence interval.

## 3. Results

Completed responses were received from 292 respondents. However, only 287 responses were used for the final analysis because of the missing data. This study shows that 50.9 percent of respondents were in age group of middle adolescents (i.e., 15-17 years), 36.6 percent of respondents were studying in class nine, 49.5 percent of respondents were Hindu, and 53.3 percent of respondents were Tamang. Regarding types of family and parent's education, 52.3 percent of respondents were living in nuclear family, and majority of respondent's father (81.9%) and more than half of respondent's mother (54.4%) were literate. Related to parent's occupation, majority of respondent's father (71.3%) were farmer and nearly half of respondent's mother (48.8%) were involving in household work. Most of respondent's parents (92.7%) were married.

Table 1 displays that majority of respondents (87.1%) did not have significant psychosocial impairments and only 12.9 percent of respondents had significant psychosocial impairment.

Table 2 displays that regarding internalizing problems, 44.6 percent of respondents had significant impairment in internalizing problems. Related to attention deficit hyperactive disorder (ADHD), 25.8 percent of respondents had significant impairment in ADHD. Regarding externalizing problems, only 4.2 percent of respondents had significant problems with conduct.

Table 3 shows that there is association between age group and psychosocial problems (*X*^2^ = 11.423,* p* = .003) and there is no association of respondents' school grade, religion, and ethnicity with psychosocial problems.

Table 4 reveals that there is association between respondents' parent's marital status and psychosocial problems (*X*^2^ = 4.960,* p* = .026). However, there is no association of respondents' types of family, father's educational status, mother's educational status, father's occupational status, and mother's occupational status with psychosocial problems.

## 4. Discussion

The present study showed that 12.9 percent of respondents had psychosocial problems. A previous cross-sectional study in Hetauda, Nepal, showed that 134 (17.03%) were suffering from psychosocial problem [11]. Another descriptive comparative study in Bharatpur also revealed that school children of nonworking mothers 11.7% had slightly more psychosocial problems than working mothers 8.3% [15]. A similar finding revealed a cross-sectional study in Pune which showed that 328 (15.2%) children were found to be at risk of psychosocial problems [16]. Findings are shown by a cross-sectional study in Muzaffarnagar, India, which revealed that the overall prevalence of psychosocial problems among adolescent was found to be 41.43% [8]. Another study in Dehradun, India, revealed the psychosocial problems among children (40.5%) [9]. Yet another study findings revealed by a cross-sectional study conducted in Dehradun which showed that the overall prevalence of psychosocial problems among the adolescents was found to be 31.2%, among them [10]. Differences in prevalence may be associated with respondent's contextual differences in national and international settings. Therefore, proper action should be taken for addressing these issues.

The current study showed the internalizing problems (44.6%), ADHD (25.8%), and externalizing problems (4.2%) among respondents. Findings of previous descriptive comparative study in Bharatpur showed that internalizing problems were found equal (13.3%) in children of working and nonworking mothers whereas attention problems were slightly higher in the school children of working mothers (11.7%) than school children of nonworking mothers (10.0%). Externalizing problems were found slightly higher in the respondents of nonworking mothers (8.3%) than children of working mothers (5.0%) [15]. Findings of another study in urban and rural areas of Dehradun showed that anxiety and conduct disorders were more common among adolescents in rural area (11.0%  and 13.0%, respectively) while depression was more common among adolescents in urban area (26.0%) [9]. Respondent's contextual differences in national and international settings might influence for this dissimilarity in findings.

The present study yielded that there is association of age group (*X*^*2*^ = 11.423,* p* = .003) and parent's marital status (*X*^*2*^ = 4.960,* p* = .026) with psychosocial problems among respondents. Similar findings which were revealed by a cross-sectional study in Hetauda, Nepal, revealed that age was significantly related to psychosocial problem. Among family factors, frequency of family dispute was highly associated with psychosocial problem [11]. A cross-sectional study in Pune also showed a similar finding that statistically significant difference was observed as per age group [16]. However, the present study revealed that there is no association of school grade, religion, ethnicity, types of family of respondents, father's educational status, mother's educational status, father's occupational status, and mother's occupational status, with psychosocial problems among respondents. Contradictory finding was revealed by a cross-sectional study in Pune which showed that statistically significant difference was observed as per class of student and similar finding was also revealed by this study that no significant difference was observed as per the type of family [16].

The considerably such prevalence of psychosocial problems among adolescents suggests the vulnerability of school going adolescents. Therefore, these findings indicate a need for national survey and launch awareness programme for preventing psychosocial problems.

## 5. Conclusions

The prevalence of psychosocial problems (internalizing problems, ADHD, and externalizing problems) is evident among school going adolescent. Adolescents' age group and parent's marital status are associated with psychosocial problems. Therefore, health care policy maker and school authority should create awareness program on psychosocial problems among adolescents, develop strategies for health promotion of adolescents, and plan for prevention of psychosocial problems among adolescents.

## 6. Limitations of This Study

This study adopted cross-sectional descriptive design covering three schools of Jarsingh Pauwa of Shankarapur Municipality, Kathmandu; therefore, the findings of this study may not be generalizable to all the school going adolescents of Nepal. Adolescents were asked about their problems that may be best fitted to them in the past days. Therefore, there may be a chance of recall bias. As this was a cross-sectional study, no causal relationship could be inferred. This study did not analyze the association between gender and psychosocial problems. Therefore, this study recommends for the replication of this study on a larger sample, covering a wider geographical area and gender-based analysis for better generalization to all school adolescents.

## Figures and Tables

**Table 1:** Psychosocial problems of respondents.

		n = 287

Characteristics	Frequency	Percent

Significant Psychological Impairment		
No	250	87.1
Yes	37	12.9

*Note. *Score of 30 or higher = significant psychological impairment (yes).

**Table 2:** Categorization of respondents' psychosocial problems.

		n = 287

Characteristics	Frequency	Percent

Internalizing Problems (Anxiety and /or Depression)		
No Impairment	159	55.4
Significant Impairment	128	44.6
Attention Deficit Problems		
No Impairment	213	74.2
Significant Impairment	74	25.8
Externalizing Problems		
No Problem	275	95.8
Significant Problems with Conduct	12	4.2

*Note.* Internalizing Problem = children with scores of 5 or higher (significant impairment). Attention Deficit Hyperactive Disorder and Externalizing Problem = children with scores 7 or higher (significant impairment).

**Table 3:** Association of respondents' age group, school grade, religion, and ethnicity with psychosocial problems.

						n = 287

Characteristics	Psychosocial Problems				*X* ^2^ Value	*p*-value
	No		Yes			
	*N*	(%)	*N*	(%)		

Age Group^a^						
Early Adolescent (12-14 years)	99	95.2	5	4.8		
Middle Adolescent (15-16 years)	123	84.2	23	15.8		
Late Adolescent (17-19 years)	28	75.7	9	24.3	11.423	.003*∗*
School Grade						
8 Class	79	90.8	8	9.2		
9 Class	85	81.0	20	19.0		
10 Class	86	90.5	9	9.5	5.590	.061
Religion						
Hindu^b^	129	90.8	13	9.2		
Bouddha and Others^c^	121	83.4	24	16.6	3.495	.062
Ethnicity						
Tamang	129	84.3	24	15.7	2.278	.131
Others^bd^	121	90.3	13	9.7		

*Note*. a = age group was categorized according to (Jahan & Shakil, 2015) [14]. b = reference category. c = Muslim and Christian. d = Bramhan, Chhetri, and Newar. *∗* = *p*-value significant at ≤ .05 level.

**Table 4:** Association of respondents' types of family, parent's educational status, occupational status, and marital status with psychosocial problems.

						n = 287

Characteristics	Psychosocial Problems				*X* ^2^ Value	*p*-value
	Absent		Present			
	*N*	(%)	*N*	(%)		

Types of Family						
Nuclear^a^	135	(90.0)	15	(10.0)		
Others^d^	115	(83.9)	22	(16.1)	2.340	.126
Father's Educational Status						
Literate	201	(85.5)	34	(14.5)	2.869	.090
Illiterate^a^	49	(94.2)	3	( 5.8)		
Mother's Educational Status						
Literate^a^	136	(87.2)	20	(12.8)		
Illiterate	114	(87.0)	17	(13.0)	0.002	.969
Father's Occupational Status (n = 275)						
Farmer^a^	175	(89.3)	21	(10.7)		
Others^b^	67	(84.8)	12	(15.2)	1.068	.301
Mother's Occupational Status (n = 284)						
Household Work	120	(85.7)	20	(14.3)		
Farmer	90	(88.2)	12	(11.8)		
Others^c^	37	(88.1)	5	(11.9)	0.386	.825
Parent's Marital Status						
Married	235	(88.3)	31	(11.7)		
Others^e^	15	(71.4)	6	(28.6)	4.960	.026*∗*

*Note*. a = reference category. b = business, service holder, laborer, and household work. c = business, service holder, and laborer. d = joint and extended family. e = widow, widower, divorced, and married but separated. *∗* = *p*-value significant at ≤ .05 level.

## Data Availability

The datasets used and/or analyzed during the current study will be available from the corresponding author on reasonable request. Individual responses will not be shared.

## Abbreviations

ADHD: Attention Deficit Hyperactive Disorder
Y-PSC: Youth Report of Pediatric Symptom Checklist
NHRC: Nepal Health Research Council
SPSS: Statistical Package for Social Sciences.
